# Association between novel inflammatory biomarkers SII, SIRI, and obesity in sedentary adults: NHANES 2007–2020

**DOI:** 10.1038/s41598-025-08121-z

**Published:** 2025-07-01

**Authors:** Yaoyao Lin, Jindong Sun, Suxia Fang, Cairong Li, Xiaojing Yang, Hong Yuan, Zhi Zhang

**Affiliations:** 1 Department of Cardiovascular, First People’s Hospital of Linping District, Hangzhou, 311100 Zhejiang P R China; 2 School of Pharmaceutical Science, Zhejiang Chinese Medical University, No. 548, Binwen Road, Binjiang District, Hangzhou, 310053 Zhejiang P R China

**Keywords:** Sedentary behavior, Systemic immune-inflammation index (SII), Systemic inflammation response index (SIRI), Obesity, NHANES, Predictive markers, Obesity

## Abstract

Sedentary behavior is prevalent in modern society and is closely associated with various chronic diseases, including obesity. The Systemic Immune-Inflammation Index (SII) and the Systemic Inflammation Response Index (SIRI), as novel inflammatory biomarkers, may play an important role in predicting obesity risk. This study utilized data from the 2007–2020 National Health and Nutrition Examination Survey (NHANES) to analyze the relationship between sedentary behavior, SII, SIRI, and obesity through multivariable logistic regression models. The study found that sedentary time was significantly associated with levels of SII and SIRI. In participants with sedentary time (ST) ≥ 5 h, the median values of SII and SIRI were 453.14 and 0.99, respectively, compared to participants with sedentary time < 5 h, whose median values of SII and SIRI were 437.82 and 0.91. The AUC value of lnSII was 0.70, indicating a high accuracy in predicting obesity risk, while the AUC value of SIRI was 0.55. Additionally, the proportion of obesity in the group with sedentary time ≥ 5 h was significantly higher than that in the group with sedentary time < 5 h, further emphasizing the association between sedentary behavior and obesity.This study demonstrates that sedentary behavior is associated with obesity risk through its impact on SII and SIRI levels, highlighting the potential importance of lifestyle changes, particularly reducing sedentary time, in controlling obesity. These findings support the application of SII and SIRI in obesity risk assessment, and future research is recommended to explore the potential of these markers in different populations.

**Supplementary Information:**

The online version contains supplementary material available at 10.1038/s41598-025-08121-z.

## Introduction

Sedentary behavior, characterized by extremely low energy expenditure, has become increasingly prevalent in modern society and has emerged as a significant global public health issue^1^. Studies have shown that sedentary behavior is closely associated with various chronic diseases, including metabolic syndrome, type 2 diabetes, cardiovascular disease (CVD), and obesity^2^. Obesity, as a global epidemic, has become one of the major factors affecting public health. Its pathogenesis is complex and involves genetic factors, appetite regulation, physiological mechanisms, insulin resistance, and chronic low-grade inflammation^3^. Sedentary behavior not only leads to an energy surplus by reducing total energy expenditure, but may also promote inflammatory responses that contribute to fat accumulation and metabolic disturbances^4^. Related studies have found that prolonged sedentary behavior is significantly associated with increased levels of inflammatory markers, such as C-reactive protein (CRP) and Interleukin-6 (IL-6), suggesting that inflammation may be an important mediator between sedentary behavior and obesity^5^.

In recent years, the importance of inflammatory biomarkers in disease risk prediction has gradually been recognized. The Systemic Immune-Inflammation Index (SII) is calculated as platelet count × neutrophil count / lymphocyte count, while the Systemic Inflammation Response Index (SIRI) is calculated as neutrophil count × monocyte count / lymphocyte count. These indices integrate key components of the immune system—SII reflects thrombotic (platelets), innate (neutrophils), and adaptive (lymphocytes) immunity, while SIRI captures the balance between pro-inflammatory and regulatory responses through neutrophils, monocytes, and lymphocytes^6^. Both indices have demonstrated good predictive efficacy in the study of various chronic diseases, such as cancer, CVD, and metabolic disorders, and have gained widespread attention^6–8^. In addition, both SII and SIRI have been confirmed to be closely associated with insulin resistance and metabolic diseases such as atherosclerosis^9^.

However, studies on the association between SII, SIRI, and obesity remain limited, particularly in sedentary populations. While some research has explored the impact of sedentary behavior on inflammation^5,10^, there is still a lack of systematic analysis regarding its relationship with systemic inflammatory biomarkers (such as SII and SIRI), as well as the role of these biomarkers in predicting obesity risk. In addition, most existing studies primarily focus on the association between sedentary behavior and traditional inflammatory markers, such as CRP and IL-6^11,12^, with limited attention given to novel inflammatory markers like SII and SIRI. Furthermore, research on the relationship between inflammatory biomarkers and obesity often overlooks sedentary behavior as a key variable, resulting in an incomplete analytical framework. More importantly, most studies are based on specific populations and lack nationwide representative data, limiting the generalizability of the findings.

Notably, recent studies have begun to examine the link between these novel markers and obesity. Zhou et al. analyzed NHANES data and reported that elevated SII and SIRI levels were significantly associated with obesity in U.S. adults, with age showing a significant interaction effect^13^. In another study, Qiu et al. found that SII demonstrated a nonlinear, inverted U-shaped association with both overall and abdominal obesity, and significant interactions were observed with age, hypertension, and diabetes status^14^. While these studies contribute important insights into the potential of SII and SIRI as predictive markers of obesity, they have largely focused on the general population and have not considered the modifying role of sedentary behavior. The present study addresses this gap by specifically targeting sedentary adults and utilizing nationally representative data from NHANES (2007–2020). Through dose–response modeling, subgroup analyses, and receiver operating characteristic (ROC) curve assessments, this study aims to provide a more comprehensive understanding of the relationship between SII, SIRI, and obesity in the context of sedentary lifestyles, thereby offering new perspectives for risk stratification and preventive strategies

## Methods

### Study population

**Fig. 1:**
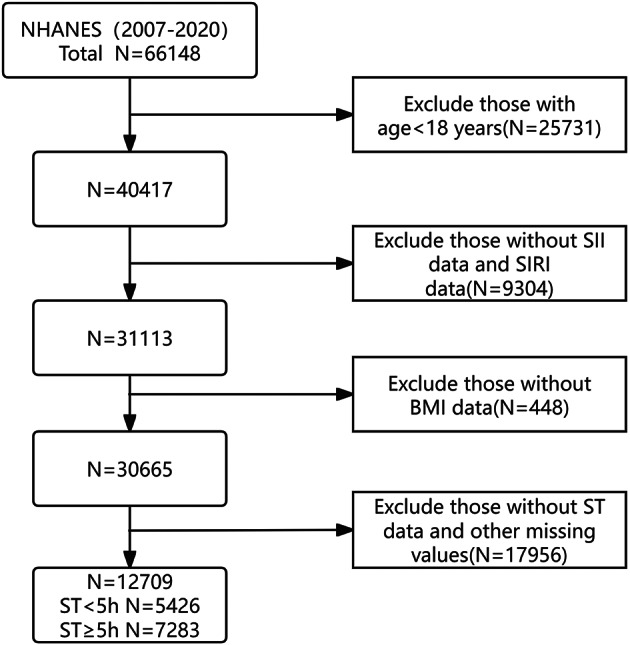
Study flowchart showing the process of participant selection.

This study is a retrospective analysis utilizing data from the National Health and Nutrition Examination Survey (NHANES). NHANES, a key project managed by the National Center for Health Statistics (NCHS) of the Centers for Disease Control and Prevention (CDC), provides representative data on the health and nutritional status of both U.S. adults and children. Its goal is to offer nationwide health insights to inform health policy and improve public health. Detailed information and data access can be found on the NHANES official website (https://www.cdc.gov/nchs/nhanes/index.html). For the NHANES cycles between 2007 and 2020, we initially included 66,148 participants. The inclusion process began by excluding 25,731 individuals under 18 years old. We then removed 9,304 participants who lacked data on the SII and SIRI. Another 448 individuals were excluded due to missing body mass index (BMI) data. Finally, 17,956 participants were excluded because they either did not report sedentary time or lacked other critical information. After applying these exclusion criteria, 12,709 participants remained for analysis, with 5,426 reporting sedentary time of less than 5 h, and 7,283 reporting 5 h or more. The participant selection process is illustrated in Fig. 1.

### Covariates data collection

Data collection was conducted by the CDC through household interviews, where standardized questionnaires were used to gather detailed information about participants, including age, gender, race, education level, socioeconomic status, smoking history (Never smoker, Ever smoker, Current smoker), alcohol consumption, and comorbidities (diabetes, hypertension, renal failure, heart failure, coronary heart disease [CHD], stroke, cancer). The present study utilized publicly available NHANES data from 2007 to 2020, which can be accessed from the official CDC website (https://www.cdc.gov/nchs/nhanes/). Laboratory measures were also rigorously tested and recorded, including lipid profiles (total cholesterol [TC], high-density lipoprotein cholesterol [HDL-C], triglycerides [TG], low-density lipoprotein cholesterol [LDL-C]), platelet count, neutrophil count, monocyte count, and lymphocyte count. All blood samples were collected at Mobile Examination Centers (MECs), where participants were instructed to fast overnight for at least 8 h prior to sample collection to ensure uniform testing conditions. Laboratory assays were performed in certified laboratories following standardized protocols outlined in the NHANES Laboratory Procedures Manual, ensuring high data quality and comparability. Further details regarding sample handling, analytic methods, and quality control procedures are available in the official NHANES Laboratory Procedures Manual published by the CDC (https://wwwn.cdc.gov/nchs/nhanes/Default.aspx).

Smoking history was classified based on whether participants had smoked at least 100 cigarettes in their lifetime and whether they currently smoked. Categories included never smoker (smoked < 100 cigarettes in lifetime), former smoker (smoked ≥ 100 cigarettes in lifetime but currently does not smoke), and current smoker. Individuals who consumed at least 12 alcoholic drinks per year were classified as alcohol consumers. The poverty-to-income ratio (PIR) was categorized as high (PIR > 3.5) or low (PIR ≤ 3.5). BMI was calculated as weight (kg) divided by the square of height (m²), with classification into non-obese (< 30.0 kg/m²) and obese (≥ 30.0 kg/m²). Diabetes was defined based on self-reported physician diagnosis or the use of insulin or glucose-lowering medications. Hypertension was defined by self-reported diagnosis, repeated elevated blood pressure measurements, or current antihypertensive treatment. Other comorbidities, including renal failure, heart failure, CHD, stroke, and cancer, were identified based on self-reported physician diagnoses.

### Definition of SII and SIRI

SII is calculated as the platelet count multiplied by the neutrophil count, divided by the lymphocyte count. SIRI is calculated as the neutrophil count multiplied by the monocyte count, divided by the lymphocyte count. SII values underwent logarithmic transformation (lnSII) to correct for right skewness^7^.

### Statistical analysis

The SII was log-transformed (lnSII) prior to analysis due to its right-skewed distribution. While normality is not required for logistic regression, transformation of highly skewed predictors can help reduce the impact of extreme values and improve model stability. The transformed variable was used consistently in all analyses. Participants were grouped into four categories (Q1, Q2, Q3, Q4) based on the quartiles of lnSII and SIRI, with Q1 serving as the reference group. Continuous variables are presented as mean ± standard deviation or median (interquartile range), and categorical variables are presented as frequencies and percentages. Independent samples t-test or Mann-Whitney test is used for between-group comparisons of continuous variables, and chi-square test is used for categorical variables. Univariate logistic regression was first performed to assess the crude association between each independent variable (e.g., SII, SIRI, sedentary time) and obesity risk without adjusting for confounders. In contrast, multivariate logistic regression models were further constructed to evaluate the independent effects of these variables by simultaneously adjusting for potential confounders, including age, gender, comorbidities (e.g., hypertension, diabetes), and socioeconomic factors (e.g., education, PIR). Covariates were selected based on prior literature and biological plausibility to control for potential confounding factors in the relationship between inflammatory markers and obesity. Demographic variables (e.g., age, gender, race), socioeconomic factors (e.g., education level, PIR), and behavioral characteristics (e.g., smoke, alcohol consumption) were included due to their known associations with both systemic inflammation and obesity. Clinical variables, such as blood lipid levels were included to adjust for metabolic status. To minimize bias, comorbid conditions (e.g., diabetes, hypertension, cardiovascular disease) were adjusted for in sensitivity analyses. To ensure model validity, multicollinearity among predictor and control variables was assessed using variance inflation factors (VIF). All VIF values were below 5, indicating no significant multicollinearity. Both univariate and multivariate logistic regression analyses were conducted to explore the relationship between inflammatory markers and obesity in sedentary individuals. Odds ratios (OR) and 95% confidence intervals (CI) were calculated as effect estimates. To determine the independent predictive value of lnSII and SIRI, three progressively adjusted models were employed. Model I was unadjusted, Model II controlled for age and gender, while Model III included further adjustments for hypertension, diabetes, race, education level, PIR, heart failure, CHD, stroke, cancer, smoking, and alcohol consumption. Some comorbidities, such as diabetes, hypertension, and cardiovascular conditions, may partially lie on the causal pathway between inflammation and obesity. Therefore, to avoid potential over-adjustment, these variables were only included in the fully adjusted model (Model III) and in sensitivity analyses, allowing comparison with models that did not adjust for these conditions. To better clarify the connection between lnSII, SIRI, and obesity risk, smoothing curve fitting was utilized to address potential non-linear relationships. In cases where non-linearity was observed between the indices and OR, we assessed various threshold values, selecting the one that maximized the likelihood ratio as the turning point. The relationship between lnSII, SIRI, and OR was further examined on both sides of this threshold. Logistic regression models were chosen because the outcome variable (obesity) was binary. Key model assumptions, including the absence of multicollinearity and approximate linearity in the logit for continuous predictors, were evaluated and found to be sufficiently met. Therefore, logistic regression was considered an appropriate and interpretable method for the main analysis. Additionally, ROC curves were employed to evaluate the effectiveness of lnSII and SIRI in predicting obesity risk among sedentary individuals. Finally, subgroup analyses were performed to examine the potential variability between different groups, including factors such as race, education level, PIR, hypertension, diabetes, CHD, heart failure, stroke, cancer, smoking, and alcohol use. Interaction terms were incorporated to assess heterogeneity across subgroups. All statistical tests were two-tailed, with a significance level set at *P* < 0.05. All statistical analyses were performed using R software (version 4.2.2; R Foundation for Statistical Computing, Vienna, Austria).

## Results

As shown in Table 1, a total of 12,709 participants were included in this study. Among them, 5,426 individuals had a sedentary time of less than 5 h (ST < 5 h), and 7,283 individuals had a sedentary time of 5 h or longer (ST ≥ 5 h). Nonparametric tests between the two groups showed that the mean age of the longer sedentary time group (ST ≥ 5 h) was 51.00 (35.00, 65.00) years, significantly higher than that of the shorter sedentary time group (50.00 (35.00, 63.00) years, *P* < 0.05). The mean value of the SII in the longer sedentary time group was 453.14 (322.34, 651.44) (×1000 cells/µl), which was higher than that in the shorter sedentary time group (437.82 (315.00, 619.37) (×1000 cells/µl), *P* < 0.001). Similarly, the SIRI was also higher in the longer sedentary time group (0.99 (0.65, 1.49) (×1000 cells/µl) vs. 0.91 (0.64, 1.34) (×1000 cells/µl), *P* < 0.001).Regarding body mass index, the obesity rate in the sedentary group was higher than that in the shorter sedentary time group (38.48% vs. 32.84%, *P* < 0.001). Furthermore, the group with longer sedentary time had higher prevalences of hypertension (41.77% vs. 35.68%, *P* < 0.001), diabetes (11.17% vs. 8.71%, *P* = 0.004), heart failure (2.93% vs. 2.11%, *P* = 0.016), CHD (4.12% vs. 2.86%, *P* = 0.001), and cancer (11.47% vs. 8.02%, *P* < 0.001) compared to the group with shorter sedentary time. Socioeconomic factors, such as education level, were also more favorable in the longer sedentary time group (87.48% with high school education or higher vs. 78.22%, *P* < 0.001). Descriptive statistics of all numerical variables (mean, median, and standard deviation) are presented in Supplement Table S1.

**Table 1:** Baseline characteristics according to ST group.

Variable	ST < 5 h (*n* = 5426)	ST > = 5 h (*n* = 7283)	*P* Value
Age, M (Q_1_, Q_3_) (years)	50.00(35.00,63.00)	51.00(35.00,65.00)	0.002
SII, M (Q_1_, Q_3_) (1000 cells/µl)	437.82(315.00,619.37)	453.14(322.34,651.44)	< 0.001
LnSII, M (Q_1_, Q_3_) (1000 cells/µl)	6.08(5.75,6.43)	6.12(5.78,6.48)	< 0.001
SIRI, M (Q_1_, Q_3_) (1000 cells/µl)	0.91(0.64,1.34)	0.99(0.65,1.49)	< 0.001
TC, M (Q_1_, Q_3_) (mmol/L)	4.89(4.22,5.61)	4.78(4.14,5.51)	< 0.001
HDL-C, M (Q_1_, Q_3_) (mmol/L)	1.34(1.11,1.63)	1.32(1.09,1.60)	< 0.001
TG, M (Q_1_, Q_3_) (mmol/L)	1.13(0.78,1.64)	1.13(0.79,1.66)	0.300
LDL-C, M (Q_1_, Q_3_) (mmol/L)	2.90(2.30,3.52)	2.82(2.25,3.44)	< 0.001
Gender, n (%)			0.643
Male	2670 (49.01)	3536 (48.47)	
Female	2756 (50.99)	3747 (51.53)	
Diabetes, n (%)			0.004
No	4705 (91.29)	6209 (88.83)	
Yes	721 (8.71)	1074 (11.17)	
Hypertension, n (%)			< 0.001
No	3267 (64.32)	3963 (58.23)	
Yes	2159 (35.68)	3320 (41.77)	
Race, n (%)			< 0.001
Mexican American	1113 (13.10)	713 (5.62)	
Non-Hispanic White	1958 (61.89)	3474 (72.30)	
Non-Hispanic Black	1090 (10.50)	1583 (10.03)	
Other	1265 (14.52)	1513 (12.05)	
Education, n (%)			< 0.001
Below high school	1696 (21.78)	1306 (12.52)	
High School or above	3730 (78.22)	5977 (87.48)	
PIR, n (%)			< 0.001
Poor (PIR ≤ 3.5)	2610 (37.83)	2815 (28.15)	
Not poor (PIR > 3.5)	2816 (62.17)	4468 (71.85)	
Renal failure, n (%)			0.106
No	5270 (97.74)	7012 (97.19)	
Yes	156 (2.16)	271 (2.73)	
Heart failure, n (%)			0.016
No	5287 (97.89)	7001 (97.07)	
Yes	139 (2.11)	282 (2.93)	
CHD, n (%)			0.001
No	5249 (97.14)	6927 (95.88)	
Yes	177 (2.86)	356 (4.12)	
Stroke, n (%)			0.157
No	5249 (97.29)	6950 (96.69)	
Yes	177 (2.71)	333 (3.31)	
Cancer, n (%)			< 0.001
No	5017 (91.98)	6459 (88.53)	
Yes	409 (8.02)	824 (11.47)	
Smoke, n (%)			0.013
Never smoker	3088 (55.03)	3951 (55.74)	
Ever smoker	1247 (24.09)	1887 (26.22)	
Current smoker	1091 (20.88)	1445 (18.04)	
Alcohol, n (%)			0.005
No	1322 (21.25)	1513 (18.03)	
Yes	4104 (78.75)	5770 (81.97)	
BMI, n (%)			< 0.001
Non-Obesity (BMI < 30 kg/m^2^)	3527 (67.16)	4336 (61.52)	
Obesity (BMI ≥ 30 kg/m^2^)	1899 (32.84)	2947 (38.48)	

Data are presented as n (%)

M: Median, Q₁: 1st Quartile, Q₃: 3st Quartile.

*SII* Systemic Immune-Inflammation Index,* SIRI* Systemic Inflammation Response Index,* lnSII* The natural logarithm of the systemic immune inflammatory index,* TC* total cholesterol,* HDL-C* high-density lipoprotein,* TG* triglyceride,* LDL-C* Low density lipoprotein-cholesterol,* BMI* body mass index (calculated as weight in kilograms divided by height in meters squatted),* PIR* ratio of family income to poverty,* CHD* coronary heart disease, Smoke Never (≥ 100 cigarettes/lifetime and currently not smoking), or current smoker (> 100 cigarettes/lifetime and currently smoking some days or every day);,

P value indicates comparison of means or proportions among two groups.

Table 2 presents the results of univariate and multivariate logistic regression analyses regarding the impact of various variables on obesity risk in individuals with sedentary time exceeding 5 h. In both univariate and multivariate analyses, females showed a significantly higher obesity risk compared to males (*OR* = 1.30, 95% *CI*: 1.13–1.50, *P* < 0.001; *OR* = 1.22, 95% *CI*: 1.04–1.43, *P* < 0.05). Both the lnSII and the SIRI were significantly associated with an increased risk of obesity in the univariate analysis, with OR of 1.62 (95% *CI*: 1.42–1.83, *P* < 0.001) and 1.16 (95% *CI*: 1.08–1.24, *P* < 0.001), respectively. However, after multivariate regression, the relationship between SIRI and obesity reversed, with higher SIRI levels being associated with a decreased obesity risk (*OR* = 0.88, 95% *CI*: 0.81–0.95, *P* < 0.05). For individuals with diabetes and hypertension, univariate analysis revealed a significantly higher obesity risk compared to those without these conditions. Diabetic individuals had an OR of 3.17 (95% *CI*: 2.54–3.94, *P* < 0.001), and hypertensive individuals had an OR of 2.34 (95% *CI*: 2.06–2.66, *P* < 0.001). Multivariate analysis continued to show significant associations for both conditions (*OR* = 2.43, 95% *CI*: 1.89–3.12, *P* < 0.001; OR = 2.33, 95% *CI*: 2.03–2.68, *P* < 0.001). In terms of race, non-Hispanic whites showed a significantly lower obesity risk compared to Mexican Americans, both in univariate analysis (*OR* = 0.64, 95% *CI*: 0.54–0.76, *P* < 0.001) and multivariate analysis (*OR* = 0.62, 95% *CI*: 0.52–0.75, *P* < 0.001). The obesity risk in non-Hispanic blacks did not show significant differences. Individuals of other races also exhibited a lower risk in both analyses (univariate *OR* = 0.44, 95% *CI*: 0.36–0.54, *P* < 0.001; multivariate *OR* = 0.43, 95% *CI*: 0.34–0.53, *P* < 0.001). Having a high school education or higher was associated with a lower obesity risk (univariate *OR* = 0.70, 95% *CI*: 0.59–0.83, *P* < 0.001). The PIR indicated in the univariate analysis that individuals above the poverty threshold had a lower obesity risk (*OR* = 0.80, 95% *CI*: 0.70–0.92, *P* < 0.05). Chronic conditions such as kidney failure and heart disease also influenced obesity risk. Kidney failure patients had a lower obesity risk in the univariate analysis (*OR* = 0.71, 95% *CI*: 0.51–0.97, *P* < 0.05), while heart failure and CHD patients exhibited an increased obesity risk (*OR* = 2.14, 95% *CI*: 1.50–3.05, *P* < 0.001; *OR* = 1.47, 95% *CI*: 1.10–1.96, *P* < 0.05). Lifestyle factors, such as smoking and alcohol consumption, were also associated with obesity. Smokers had a slightly increased obesity risk in the univariate analysis (*OR* = 1.17, 95% *CI*: 1.02–1.35, *P* < 0.05), while drinkers showed a lower obesity risk (*OR* = 0.76, 95% *CI*: 0.65–0.89, *P* < 0.001).

**Table 2:** Univariate and multivariate logistic regression analysis of predictors for obesitys in the sedentary group (≥ 5 h).

Variables	Obesity Univariate	Obesity Multivariate
	OR (95%CI)*P*-value	OR (95%CI)*P*-value
Gender		
Male	1.00	1.00
Female	1.30 (1.13 ~ 1.50)^**^	1.22 (1.04 ~ 1.43)^*^
lnSII	1.62 (1.42 ~ 1.83)^**^	1.84 (1.58 ~ 2.14)^**^
SIRI	1.16 (1.08 ~ 1.24)^**^	0.88 (0.81 ~ 0.95)^*^
Diabetes		
No	1.00	1.00
Yes	3.17 (2.54 ~ 3.94)^**^	2.43 (1.89 ~ 3.12)^**^
Hypertension		
No	1.00	1.00
Yes	2.34 (2.06 ~ 2.66)^**^	2.33 (2.03 ~ 2.68)^**^
Race		
Mexican American	1.00	1.00
Non-Hispanic White	0.64 (0.54 ~ 0.76)^**^	0.62 (0.52 ~ 0.75)^**^
Non-Hispanic Black	1.14 (0.93 ~ 1.39)^#^	1.13 (0.91 ~ 1.42)^#^
Other	0.44 (0.36 ~ 0.54)^**^	0.43 (0.34 ~ 0.53)^**^
Education		
Below high school	1.00	1.00
High School or above	0.70 (0.59 ~ 0.83)^**^	0.85 (0.70 ~ 1.03)^#^
PIR		
Poor(PIR ≤ 3.5)	1.00	1.00
Not poor(PIR ≤ 3.5)	0.80 (0.70 ~ 0.92)^*^	0.90 (0.77 ~ 1.04)^#^
Renal failure		
No	1.00	1.00
Yes	0.71 (0.51 ~ 0.97)^*^	1.24 (0.83 ~ 1.85)^#^
Heart failure		
No	1.00	1.00
Yes	2.14 (1.50 ~ 3.05)^**^	1.46 (0.94 ~ 2.25)^#^
CHD		
No	1.00	1.00
Yes	1.47 (1.10 ~ 1.96)^*^	0.98 (0.70 ~ 1.39)^#^
Stroke		
No	1.00	1.00
Yes	1.43 (1.01 ~ 2.04)^*^	0.92 (0.63 ~ 1.36)^#^
Smoke		
Never smoker	1.00	1.00
Ever smoker	1.17 (1.02 ~ 1.35)^*^	1.12 (0.96 ~ 1.31)^#^
Current smoke	0.80 (0.65 ~ 1.00)^#^	0.68 (0.54 ~ 0.87)^*^
Alcohol		
No	1.00	1.00
Yes	0.76 (0.65 ~ 0.89)^**^	0.87 (0.73 ~ 1.03)^#^

*CHD* coronary heart disease,* PIR* poverty to income ratio; lnSII, the natural logarithm of the systemic immune inflammatory index,* SIRI* systemic inflammation response index,

# No significant; * *P* < 0.05; **P < 0.001.

Tables 3 and 4 analyze the relationship between lnSII and SIRI with obesity risk among 7,283 sedentary participants who reported sitting more than 5 h per day. In Table 3, after adjusting for age, gender, and a broad range of health and socioeconomic factors using three multivariate logistic regression models, a significant increase in obesity risk was observed with higher lnSII levels. In the initial model (Model I), which did not adjust for any covariates, the highest quartile of lnSII showed the greatest obesity risk (*OR* = 1.91, 95% *CI*: 1.61–2.26, *P* < 0.001), and this trend remained significant after further adjustments in the subsequent models. In the analysis presented in Table 4, each incremental increase in SIRI was significantly associated with an elevated risk of obesity, particularly in the higher quartiles where this relationship was more pronounced. Even after adjusting for age and gender in Model II and incorporating additional adjustments for health conditions and lifestyle factors in Model III, the association between higher quartile SIRI levels and a significantly increased obesity risk remained stable.

**Table 3:** Associations between the natural logarithm of the systemic immune inflammatory index(lnSII) and obesity in sedentary population.

Variables	Model I		Model II		Model III	
	OR (95%CI)	*P*-value	OR (95%CI)	*P*-value	OR (95%CI)	*P*-value
lnSII						
Q1	1.00		1.00		1.00 (Reference)	
Q2	1.19 (1.00 ~ 1.42)	0.058	1.17 (0.98 ~ 1.40)	0.086	1.17 (0.97 ~ 1.41)	0.111
Q3	1.61 (1.38 ~ 1.88)	< 0.001	1.55 (1.33 ~ 1.81)	< 0.001	1.56 (1.33 ~ 1.83)	< 0.001
Q4	1.91 (1.61 ~ 2.26)	< 0.001	1.80 (1.52 ~ 2.12)	< 0.001	1.69 (1.43 ~ 2.01)	< 0.001

Three multivariate logistic regression models:

Model I: no covariates were adjusted.

Model II: Model I + age, and gender were adjusted.

Model III: Model II + Hypertension, diabetes, Race, Education, PIR, Heart failure, CHD, Stroke, Cancer, Smoke, Alcohol were adjusted.

**Table 4:** Associations between the systemic inflammation response index (SIRI) and obesity in sedentary population.

Variables	Model1		Model II		Model III	
	OR (95%CI)	*P*-value	OR (95%CI)	*P*-value	OR (95%CI)	*P*-value
SIRI						
Q1	1.00		1.00		1.00	
Q2	1.17 (1.01 ~ 1.36)	0.047	1.20 (1.03 ~ 1.40)	0.025	1.17 (1.00 ~ 1.36)	0.054
Q3	1.60 (1.32 ~ 1.95)	< 0.001	1.63 (1.34 ~ 1.98)	< 0.001	1.56 (1.28 ~ 1.91)	< 0.001
Q4	1.84 (1.57 ~ 2.16)	< 0.001	1.85 (1.56 ~ 2.18)	< 0.001	1.67 (1.42 ~ 1.97)	< 0.001

Three multivariate logistic regression models:

Model I: no covariates were adjusted.

Model II: Model I + age, and gender were adjusted.

Model III: Model II + Hypertension, Diabetes, Race, Education, PIR, Heart failure, CHD, Stroke, Cancer, Smoke, Alcohol were adjusted.

**Fig. 2:**
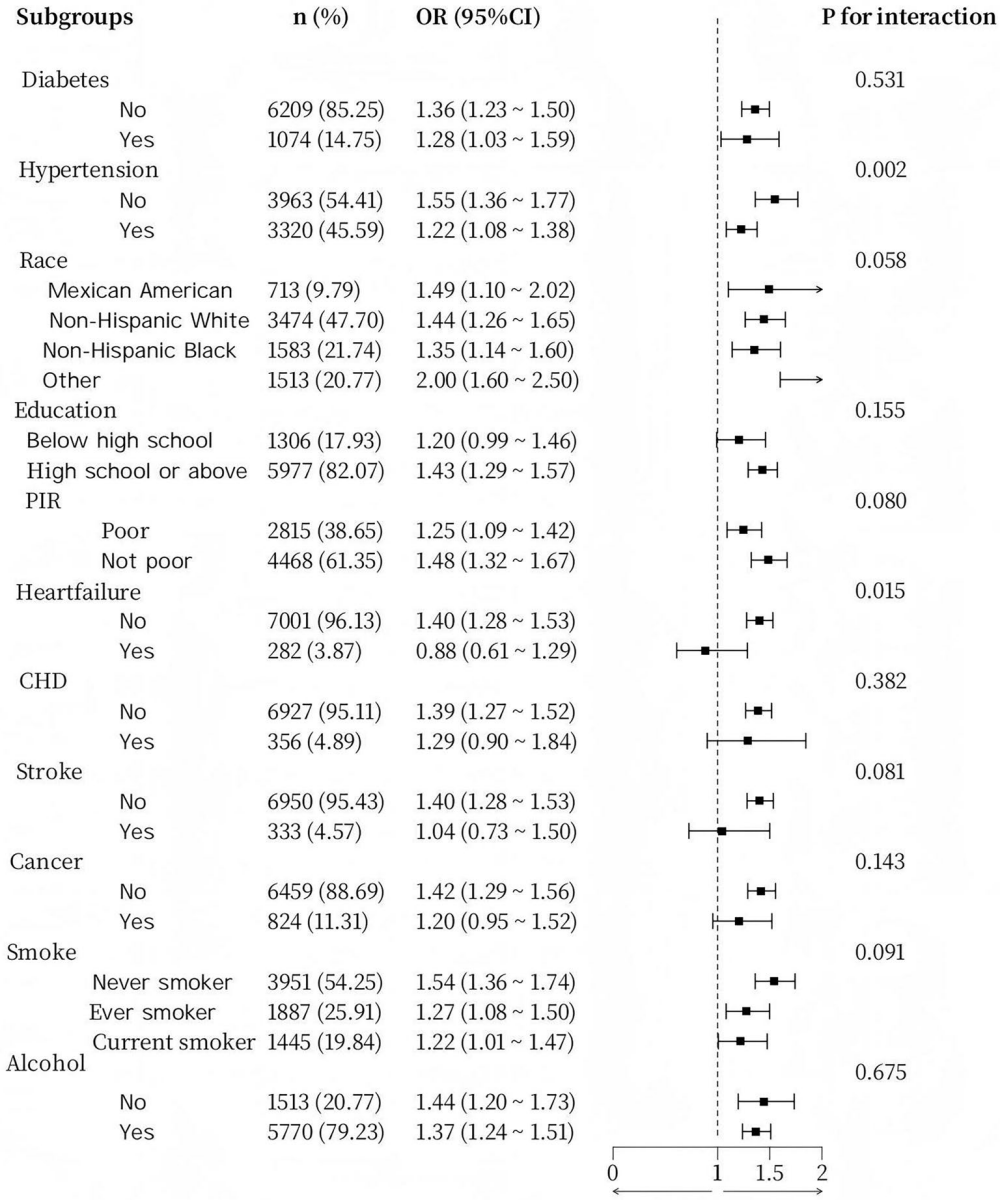
Forest plot of SII and BMI subgroups after adjusting for gender and age covariates.

**Fig. 3:**
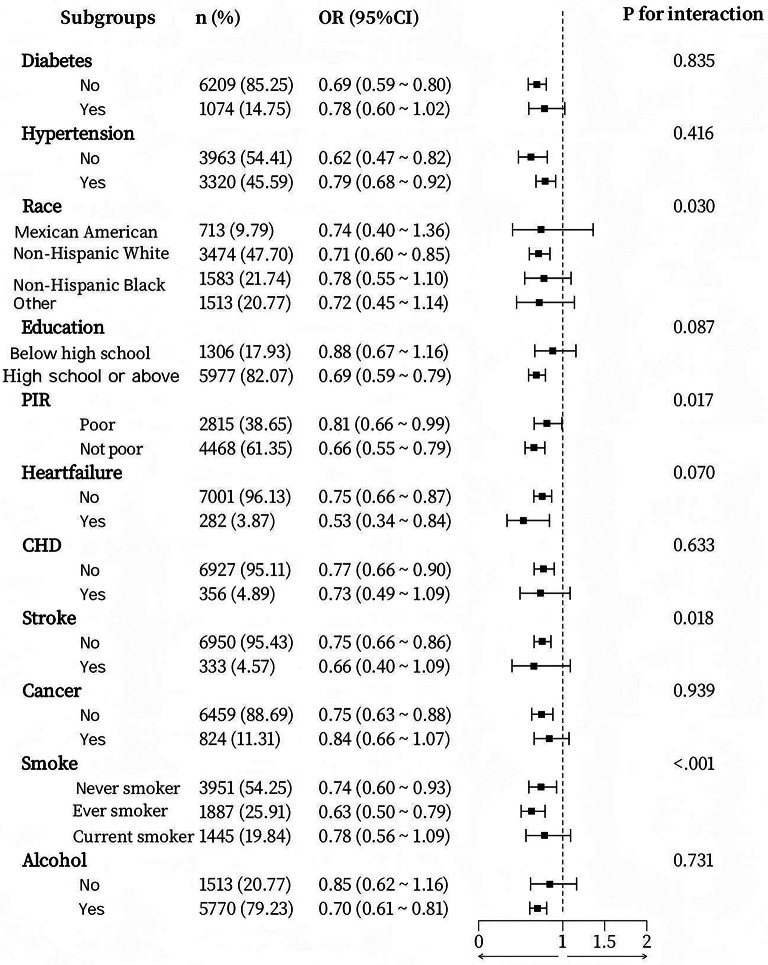
Forest plot of SIRI and BMI subgroups after adjusting for gender and age covariates.

To assess the robustness of the relationship between SII and obesity in various subgroups, we conducted a subgroup analysis. Interaction tests revealed that, after controlling for gender and age, the link between SII and obesity remained consistent across most subgroups, with no statistically significant differences observed (Fig. 2). Variables such as race, educational attainment, PIR, smoking status, alcohol consumption, diabetes, coronary artery disease, stroke, and cance did not substantially alter this association (all interaction *P* values > 0.05). In contrast, the hypertension (yes/no) subgroup exhibited a significant interaction, with hypertensive patients showing a greater likelihood of obesity (*OR* = 1.22, *P* = 0.002). Furthermore, after adjusting for gender and age, although the association between SIRI and obesity appeared to be marginally influenced by factors such as race, PIR, stroke, and smoking, no notable differences were found in most subgroups (Fig. 3), including hypertension, diabetes, education level, heart failure, coronary artery disease, cancer, and alcohol consumption. These results suggest that the relationships between SII, SIRI, and obesity are generally stable and dependable across the majority of subgroups.

**Fig. 4:**
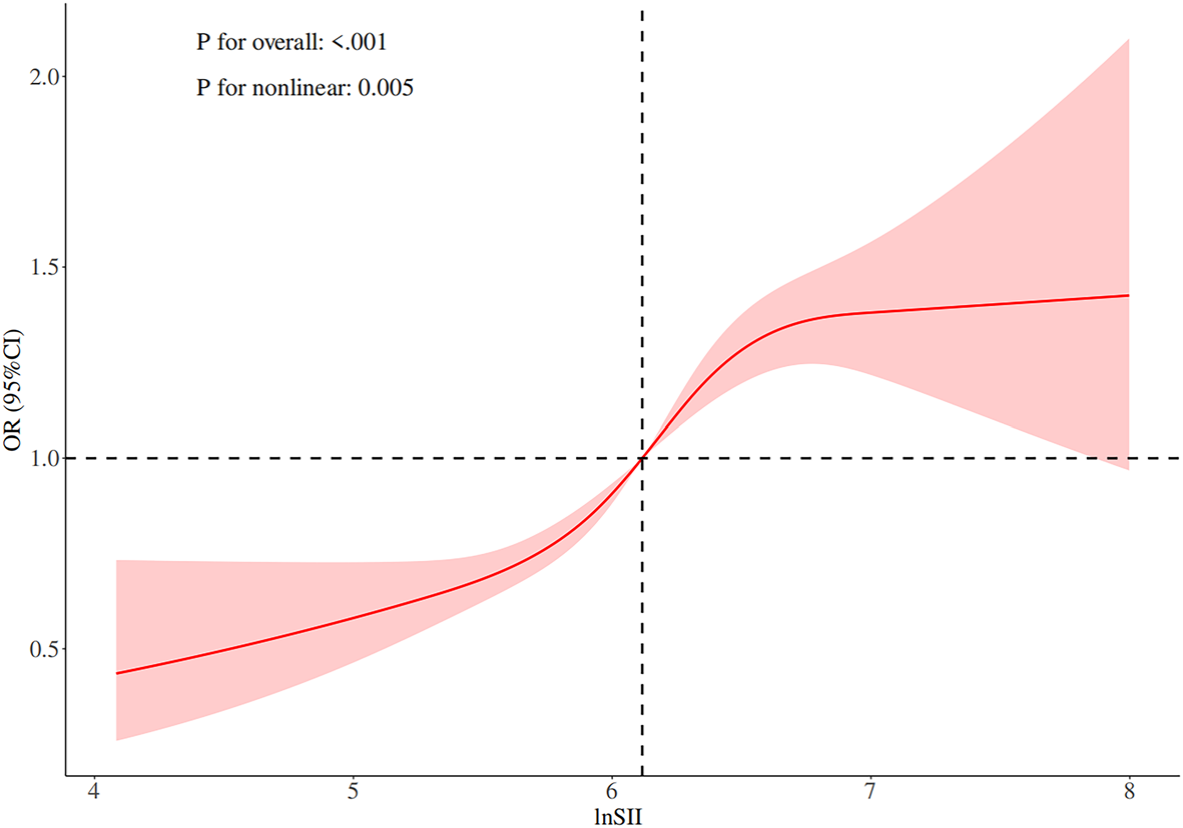
RCS curve of lnSII after adjusting for gender and age.

**Fig. 5:**
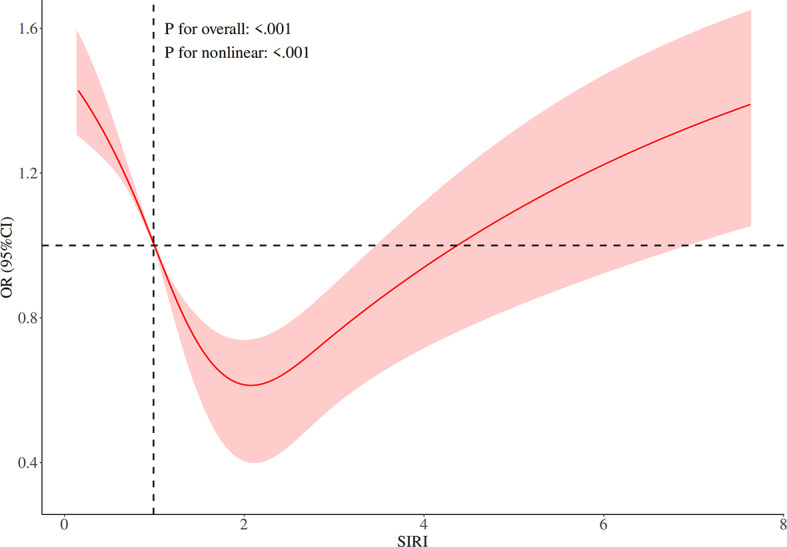
RCS curve of SIRI after adjusting for gender and age.

Figures 4 and 5 display the dose-response curves between the lnSII, SIRI, and specific health outcomes such as obesity. From Fig. 4, it can be seen that when lnSII is below 6, the associated health risks are relatively low, but beyond this point, the risks increase significantly, demonstrating a nonlinear relationship between lnSII and health risks. This pattern is statistically significant (overall model *P* < 0.001, nonlinear *P* = 0.005), revealing the intermediate value of lnSII as a turning point for health risks. In Fig. 5, when SIRI is low (below approximately 2), a gradual reduction in health risks is observed; however, once SIRI exceeds 2, the risk begins to rise significantly and continues to increase with further elevation of SIRI. Both the overall and nonlinear components of this relationship are statistically significant (*P* < 0.001), indicating a complex and significant nonlinear association between SIRI and health outcomes.

**Fig. 6:**
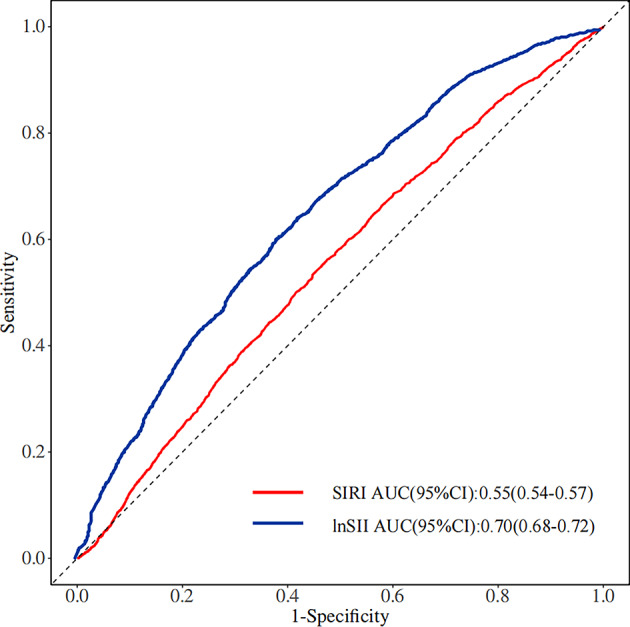
Receiver operating characteristic curve. Predictive accuracy was classified as weak (0.60–0.70), acceptable (0.70–0.80), and good discriminator (0.80–0.90), based on conventional thresholds.

We used ROC curves (Fig. 6) to assess the diagnostic performance of the lnSII and the SIRI in predicting specific health outcomes such as obesity. In the ROC analysis presented, lnSII demonstrated high predictive effectiveness, with an area under the curve (AUC) of 0.70 (95% *CI*: 0.68–0.72), indicating good predictive accuracy. In contrast, SIRI showed lower predictive performance, with an AUC of only 0.55 (95% *CI*: 0.54–0.57), which is close to random guessing (AUC = 0.50). Therefore, overall, the predictive performance of lnSII is significantly superior to that of SIRI.

**Fig. 7:**
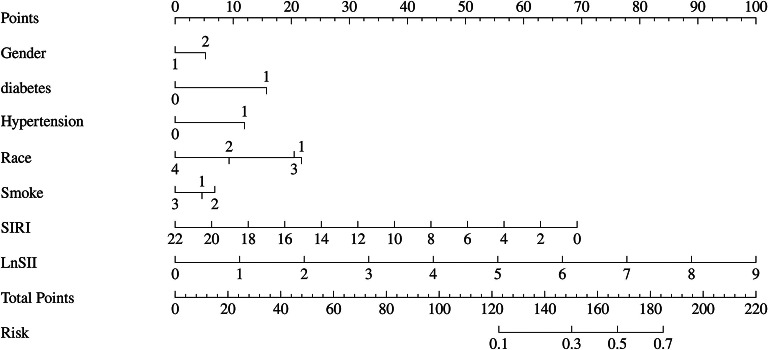
Nomogram of obesity occurrence among sedentary populations.

**Fig. 8:**
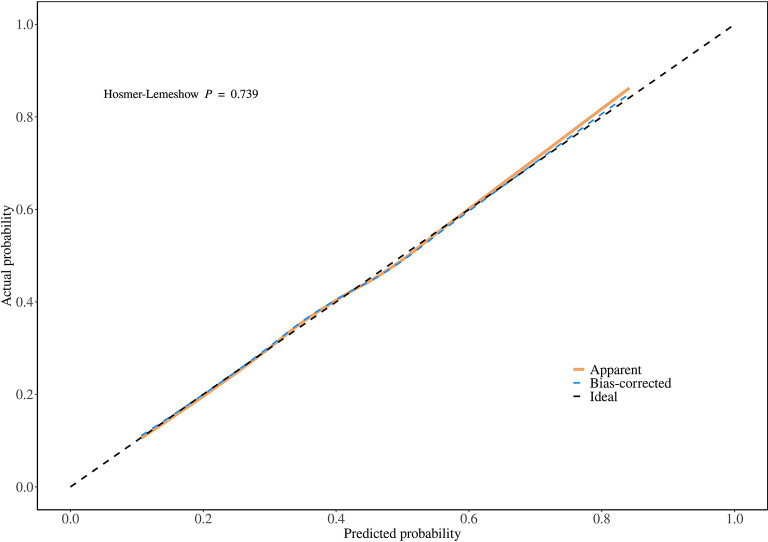
Calibration curve of the training set and Hosmer-Lemeshow test.

**Fig. 9:**
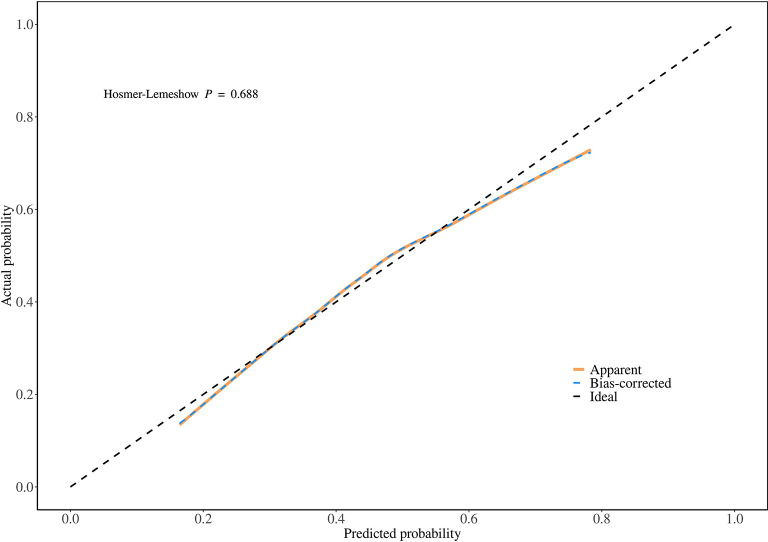
Calibration curve of the validation set and Hosmer-Lemeshow test.

Additionally, in this study, the data of sedentary populations was divided into a 70% training set and a 30% validation set. Through univariate and multivariate logistic regression analyses, seven influencing factors with significant potential in obesity diagnosis and prediction were screened out, and an obesity diagnostic nomogram for sedentary populations (Fig. 7) was constructed based on these seven variables. The Hosmer-Lemeshow test was used to evaluate the consistency between the predicted probabilities of the logistic regression model and the actual observed outcomes. The test results showed that the chi-square value of the training set was 5.18 (degrees of freedom = 8, *P* = 0.739, Fig. 8), and that of the validation set was 5.62 (degrees of freedom = 8, *P* = 0.688, Fig. 9). The P-values of both groups were greater than 0.05, indicating no statistically significant difference between the model’s predicted values and the actual observed values. The calibration curve further showed that the predicted probabilities were highly consistent with the actual probabilities along the diagonal, confirming that the model calibration had good stability and reliability. The results of this study validate the effectiveness of the model in the diagnosis and prediction of obesity in sedentary populations, providing a robust tool support for clinical decision-making and risk assessment.

**Fig. 10:**
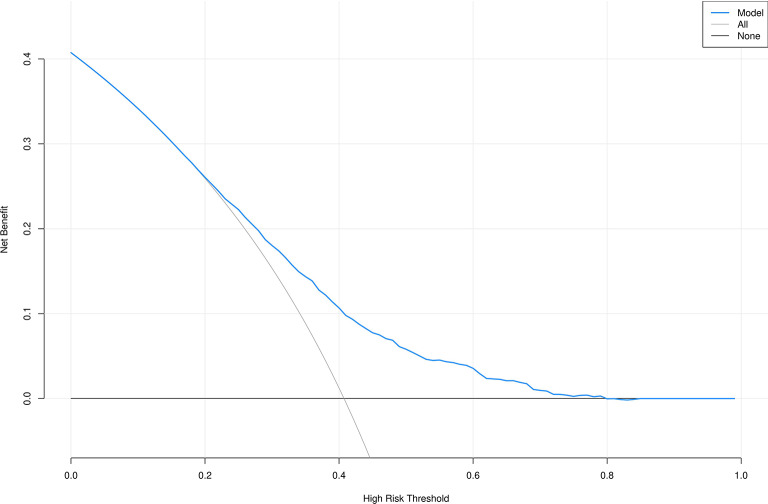
DCA curve of the training set. All: All patients received the intervention; None: All patients did not receive intervention.

**Fig. 11:**
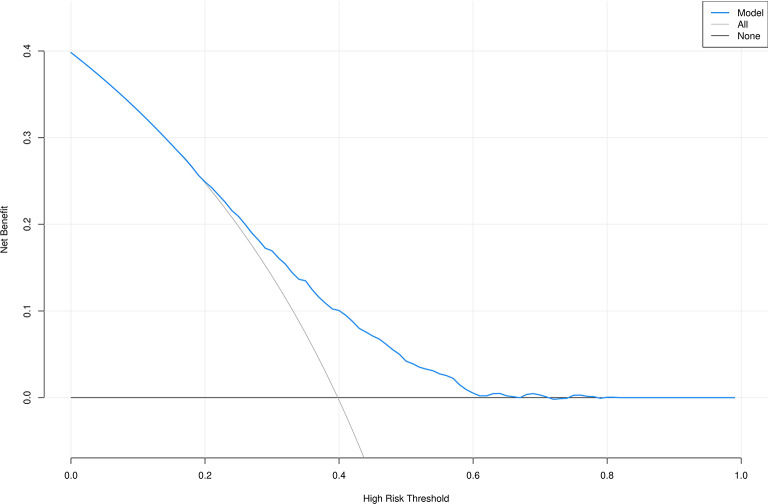
DCA curve of the validation set; All: All patients received the intervention; None:All patients did not receive intervention.

As shown in Figs. 10 and 11, the Decision Curve Analysis (DCA) plots of the predictive models for the training set and validation set were higher than the “treat all” and “treat none” curves within the threshold probability ranges of 0.2–0.8 and 0.2–0.6, respectively. This result visually demonstrates that the predictive model incorporating seven variables has significant advantages in clinical net benefit, enabling effective identification of individuals at high risk of obesity through risk stratification and providing a quantitative basis for formulating personalized clinical intervention plans. Additionally, combined with ROC curve analysis, the lnSII showed superior predictive efficacy for obesity risk compared to the SIRI, suggesting that lnSII may be a more optimal biomarker for predicting obesity risk.

## Discussion

This study, based on the NHANES database, aims to analyze the relationship between sedentary behavior, SII, SIRI, and obesity. Using nationally representative data, we comprehensively assessed the association between inflammatory biomarkers (SII, SIRI) and obesity across different sedentary time periods, and further conducted stratified analyses to explore the variable characteristics in different populations. The results indicate that sedentary behavior is not only a significant risk factor for obesity but may also contribute to its development by influencing inflammation levels. SII and SIRI, as novel inflammatory markers, show a significant association with obesity risk, with lnSII demonstrating strong predictive potential.

This study found that individuals with longer sedentary time (≥ 5 h) had significantly higher levels of SII and SIRI compared to those with shorter sedentary time (< 5 h). These findings suggest that sedentary behavior may be associated with systemic inflammation^15^. Previous studies have shown a significant association between sedentary behavior and chronic low-grade inflammation, with increased sedentary time potentially leading to elevated levels of inflammatory markers such as CRP, IL-6, and tumor necrosis factor -α (TNF-α)^16–18^. Our findings are consistent with these studies. Furthermore, we observed that individuals with longer sedentary time had a significantly higher prevalence of metabolic diseases such as hypertension and diabetes, further supporting the idea that sedentary behavior may exacerbate metabolic disorders through inflammation^19^. This finding is similar to the research by Evelien J Vandercappellen et al., which highlighted the association between sedentary behavior, low-grade inflammation (e.g., CRP), and endothelial dysfunction biomarkers, which in turn are closely related to prediabetes and metabolic disorders^20^. Additionally, our study found that individuals with longer sedentary time had higher levels of SII and SIRI compared to the control group, but the variation in SIRI was smaller than that of SII. Whether this indicates that SII has a greater advantage in assessing inflammatory and immune responses comprehensively requires further investigation in future studies.

In the dose-response analysis between the lnSII, SIRI, and obesity, this study found a significant association between both inflammatory markers and obesity risk. However, there were notable differences in their performance: lnSII showed a gradual increase in obesity risk as its level rose, exhibiting a nonlinear pattern, with the intermediate value of lnSII identified as a turning point for risk; while SIRI displayed a more complex nonlinear relationship. At lower to moderate levels of SIRI (approximately 0–2), obesity risk (*OR* value) gradually decreased, but when SIRI levels increased further (> 2), the risk of obesity significantly increased. This dose-response pattern may reflect the different biological significance of these two inflammatory markers in the association between inflammation and obesity. An increase in SII can be considered a marker of inflammation, reflecting an increase in neutrophil and platelet counts, components that are closely related to chronic inflammation associated with obesity^21,22^. Previous studies have shown that neutrophils exacerbate the chronic inflammatory state of adipose tissue by releasing reactive oxygen species (ROS) and cytokines (e.g., IL-6, TNF-α), thereby promoting the development of obesity^23^. Platelets exhibit hyperreactivity in obesity, making them more easily activated, which increases the risk of thrombosis and further promotes atherosclerosis and CVD^24^. These mechanisms partially support the potential value of SII as a comprehensive inflammatory marker in obesity risk assessment.

At lower to moderate levels, SIRI shows an inverse correlation with obesity risk, which may reflect the anti-inflammatory effect of monocytes differentiating into M2 macrophages in a lower inflammation state. This relationship shifts at higher levels of SIRI, where the association with obesity risk becomes positive once a certain threshold is crossed. This could suggest that while SIRI may help suppress obesity-related inflammation at low to moderate levels, pro-inflammatory factors may begin to dominate at higher levels, thereby increasing obesity risk. The findings of this study contrast sharply with most previous studies, which have generally reported a positive correlation between SIRI and health risks. For example, Pengbo Wang and colleagues found that higher levels of SIRI are usually associated with an increased risk of metabolic diseases and a positive correlation with CVD risk^25^. Similarly, Zhou Liu and others observed a significant positive correlation between SIRI and CVD prevalence, suggesting that SIRI could serve as an effective inflammatory marker for assessing CVD risk in obese populations^26^. This study observed a negative association between SIRI and obesity risk, which may appear counterintuitive but could have biological plausibility. First, individuals with severe obesity may develop compensatory inflammatory regulatory mechanisms, characterized by a relative reduction in circulating monocytes and neutrophils—a phenomenon known as “inflammatory exhaustion”. Second, lymphocytes, which are part of the SIRI calculation, often increase reactively in response to metabolic disturbances, potentially offsetting the pro-inflammatory effects of the numerator components. Additionally, this association was more pronounced among older adults and individuals with diabetes, suggesting that the degree of metabolic dysregulation and age may influence the relationship between inflammatory markers and obesity^27–29^. Although this finding is inconsistent with some previous studies, it aligns with reports on dynamic changes in inflammatory markers among severely obese populations, reflecting the complex and heterogeneous inflammatory characteristics of obesity. The unique nonlinear relationship in this study highlights the dual biological nature of SIRI, and future research needs to explore more deeply the dynamic changes of SIRI in different physiological and pathological states and how these changes affect the development of obesity and other diseases.

ROC curve analysis revealed differences between the SII and the SIRI in predicting obesity risk. Specifically, SII demonstrated acceptable predictive accuracy with an AUC of 0.70, while SIRI had an AUC of only 0.55, suggesting its lower effectiveness in predicting obesity risk. This finding suggests that although both SII and SIRI are indicators of systemic inflammation, their distinct cellular components may reflect different aspects of the immune-inflammatory response related to obesity. SII integrates platelet, neutrophil, and lymphocyte counts, whereas SIRI is based on the ratio of neutrophils, monocytes, and lymphocytes. This difference in cellular composition may explain the biological variation in their predictive performance. A study by Yang et al. found that SII had higher sensitivity and specificity in predicting CVD risk and performed better than traditional risk factors in forecasting cardiovascular events^30^. SII may offer a broader reflection of immune-inflammatory status, as it incorporates platelet, neutrophil, and lymphocyte counts, representing the thrombotic, innate, and adaptive immune responses, respectively, which may explain its superiority in predicting obesity risk^31^. However, existing literature typically demonstrates the effectiveness of each of these markers in predicting different health risks, with few directly comparing the predictive capabilities of SII and SIRI^6,32,33^. Therefore, the results of this study provide a new direction for future research, emphasizing the need for deeper exploration of the predictive efficacy of SII and SIRI in different diseases and health conditions, as well as a direct comparison of their performance differences.

The associations between sedentary behavior, inflammatory markers, and obesity identified in this study may be mediated through the following mechanisms: First, prolonged sedentary behavior reduces skeletal muscle contraction, leading to a significant decline in lipoprotein lipase activity, which triggers dyslipidemia and adipose tissue expansion^34^. Fat accumulation-induced tissue hypoxia can activate the NF-κB inflammatory pathway, promoting the secretion of pro-inflammatory factors such as TNF-α and IL-6 by adipocytes^15^, while also recruiting M1 macrophage infiltration, thereby amplifying the inflammatory response^35^. Second, the elevated SII and SIRI observed in this study reflect systemic immune activation in this process: adipose-derived IL-6 stimulates neutrophil production^36^, leptin and other adipokines enhance platelet activity^37^, and increased expression of vascular endothelial adhesion molecules promotes monocyte migration^38^. These changes collectively contribute to a vicious cycle of “inflammation-insulin resistance-further obesity”: inflammatory factors promote lipid accumulation by inhibiting insulin signaling pathways, while simultaneously downregulating the anti-inflammatory adiponectin^39,40^, ultimately leading to metabolic dysfunction.

This study has several strengths. First, it is based on a nationally representative sample from the NHANES, which includes a diverse population with varying characteristics such as age, gender, race, and socioeconomic status, ensuring the broad applicability of the results. Second, the study utilized novel inflammatory markers (SII and SIRI), which integrate multiple blood cell count parameters and provide a comprehensive reflection of systemic inflammation, offering a more efficient evaluation method compared to traditional inflammatory markers like CRP and IL-6. Third, the study employed multi-level statistical analyses, including dose-response and ROC curve analyses, which not only revealed the nonlinear relationship between lnSII and SIRI with obesity risk but also assessed their predictive performance, providing important scientific evidence for public health policies and early obesity screening. However, there are also some limitations. First, since the study is based on cross-sectional data from NHANES, it cannot establish causal relationships between sedentary behavior, inflammatory markers, and obesity; it only reveals correlations. Second, the study relies on self-reported questionnaires and laboratory data, which may introduce information bias or measurement error, potentially affecting the precision of the results. Third, although our fully adjusted model included comorbid conditions such as diabetes, hypertension, and heart disease, we acknowledge that these variables may act as mediators in the causal pathway linking inflammation and obesity. Their inclusion may lead to over-adjustment and potentially attenuate the estimated effects of inflammatory markers. Fourth, sedentary time was self-reported, which may introduce recall bias or misclassification issues. Although the NHANES database does not directly report validity verification of the questionnaire, we can indirectly assess the clinical predictive validity of sedentary time by reviewing other literature. The results show that even after adjusting for various variables, the association between sedentary time and sleep disorders remains significant^41,42^, which indirectly supports the validity of the questionnaire. Fifth, due to the cross-sectional design of this study, causal relationships cannot be established. It is also possible that obesity may lead to increased sedentary time and higher levels of inflammation. Therefore, longitudinal or interventional studies are needed to clarify the directionality of these associations. Another important limitation of this study is the lack of adjustment for dietary intake and overall physical activity levels, which are known to influence both systemic inflammation and obesity risk. Although NHANES collects information on these factors, the available data were either incomplete or inconsistent across survey cycles. Consequently, residual confounding cannot be ruled out, and future studies incorporating these lifestyle factors are warranted to validate our findings. Lastly, while the study adjusted for various confounding factors (such as age, gender, race, and socioeconomic status), some potential influencing factors (such as dietary habits and genetic background) were not controlled for, which might have had an unaccounted impact on the results. Future research should incorporate longitudinal data and intervention trials to further validate the role of inflammatory markers in the development of obesity and explore the effects of sedentary behavior interventions on inflammation and obesity.

## Conclusion

This study found that sedentary behavior is closely associated with increased levels of SII and SIRI, both of which may play an important role in predicting obesity risk. These findings highlight the connection between a sedentary lifestyle and obesity-related inflammation, providing a scientific basis for using SII and SIRI as potential tools for assessing and intervening in obesity. Future research should further explore the application of these markers in clinical and public health strategies, with the aim of developing more effective interventions to mitigate the impact of obesity and its associated health issues.

## Electronic supplementary material

Below is the link to the electronic supplementary material.

Supplementary Material 1

## Data Availability

Data availabilityThe datasets analysed during the current study are available in the NHANES repository, https://www.cdc.gov/nchs/nhanes/index.html.

## References

[1] Yang, L. et al. Trends in sedentary behavior among the US population, 2001–2016. *Jama* **321** (16), 1587–1597 (2019). 31012934 10.1001/jama.2019.3636 PMC6487546

[2] Patterson, R. et al. Sedentary behaviour and risk of all-cause, cardiovascular and cancer mortality, and incident type 2 diabetes: a systematic review and dose response meta-analysis. *Eur. J. Epidemiol.* **33** (9), 811–829 (2018). 29589226 10.1007/s10654-018-0380-1 PMC6133005

[3] Caballero, B. The global epidemic of obesity: an overview. *Epidemiol. Rev.* **29**, 1–5 (2007). 17569676 10.1093/epirev/mxm012

[4] Verdú, E., Homs, J. & Boadas-Vaello, P. Physiological changes and pathological pain associated with sedentary Lifestyle-Induced body systems fat accumulation and their modulation by physical exercise. *Int. J. Environ. Res. Public. Health* **18**(24). (2021). 10.3390/ijerph182413333 PMC8705491 34948944

[5] Henson, J. et al. Sedentary time and markers of chronic low-grade inflammation in a high risk population. *PLoS One*. **8** (10), e78350 (2013). 24205208 10.1371/journal.pone.0078350 PMC3812126

[6] Menyhart, O., Fekete, J. T. & Győrffy, B. Inflammation and colorectal cancer: A Meta-Analysis of the prognostic significance of the systemic Immune-Inflammation index (SII) and the systemic inflammation response index (SIRI). *Int. J. Mol. Sci.* **25**(15). (2024). 10.3390/ijms25158441 PMC11312822 39126008

[7] Jin, Z. et al. The associations of two novel inflammation indexes, SII and SIRI with the risks for cardiovascular diseases and All-Cause mortality: A Ten-Year Follow-Up study in 85,154 individuals. *J. Inflamm. Res.* **14**, 131–140 (2021). 33500649 10.2147/JIR.S283835 PMC7822090

[8] Xie, S. et al. Associations of systemic immune-inflammation index and systemic inflammation response index with maternal gestational diabetes mellitus: evidence from a prospective birth cohort study. *Chin. Med. J. (Engl)* (2024). 10.1097/CM9.0000000000003236 PMC11925413 39648043

[9] Song, Y. et al. Combination model of neutrophil to high-density lipoprotein ratio and system inflammation response index is more valuable for predicting peripheral arterial disease in type 2 diabetic patients: A cross-sectional study. *Front. Endocrinol. (Lausanne)*. **14**, 1100453 (2023). 36875480 10.3389/fendo.2023.1100453 PMC9978802

[10] Bergens, O., Nilsson, A., Papaioannou, K. G. & Kadi, F. Sedentary patterns and systemic inflammation: sex-specific links in older adults. *Front. Physiol.* **12**, 625950 (2021). 33613317 10.3389/fphys.2021.625950 PMC7892961

[11] Parsons, T. J. et al. Physical activity, sedentary behavior, and inflammatory and hemostatic markers in men. *Med. Sci. Sports Exerc.* **49** (3), 459–465 (2017). 28222056 10.1249/MSS.0000000000001113 PMC5330416

[12] Falconer, C. L. et al. Sedentary time and markers of inflammation in people with newly diagnosed type 2 diabetes. *Nutr. Metab. Cardiovasc. Dis.* **24** (9), 956–962 (2014). 24925122 10.1016/j.numecd.2014.03.009 PMC4154448

[13] Zhou, Y., Wang, Y., Wu, T., Zhang, A. & Li, Y. Association between obesity and systemic immune inflammation index, systemic inflammation response index among US adults: a population-based analysis. *Lipids Health Dis.* **23** (1), 245 (2024). 39127686 10.1186/s12944-024-02240-8 PMC11316435

[14] Qiu, L. et al. Association of systemic immune inflammatory index with obesity and abdominal obesity: A cross-sectional study from NHANES. *Nutr. Metab. Cardiovasc. Dis.* **34** (10), 2409–2419 (2024). 39069464 10.1016/j.numecd.2024.06.003

[15] Bruunsgaard, H. Physical activity and modulation of systemic low-level inflammation. *J. Leukoc. Biol.* **78** (4), 819–835 (2005). 16033812 10.1189/jlb.0505247

[16] Allison, M. A., Jensky, N. E., Marshall, S. J., Bertoni, A. G. & Cushman, M. Sedentary behavior and adiposity-associated inflammation: the multi-ethnic study of atherosclerosis. *Am. J. Prev. Med.* **42** (1), 8–13 (2012). 22176840 10.1016/j.amepre.2011.09.023 PMC3244676

[17] Arouca, A. B. et al. Diet as a moderator in the association of sedentary behaviors with inflammatory biomarkers among adolescents in the HELENA study. *Eur. J. Nutr.* **58** (5), 2051–2065 (2019). 29974229 10.1007/s00394-018-1764-4

[18] MacNeil, L. G., Tarnopolsky, M. A., Crane, J. D. & Acute exercise-induced alterations in cytokines and chemokines in the blood distinguish physically active and sedentary aging. *J. Gerontol. Biol. Sci. Med. Sci.* **76** (5), 811–818 (2021). 10.1093/gerona/glaa310 PMC8087278 33289019

[19] Pinto, A. J. et al. Physiology of sedentary behavior. *Physiol. Rev.* **103** (4), 2561–2622 (2023). 37326297 10.1152/physrev.00022.2022 PMC10625842

[20] Vandercappellen, E. J. et al. Sedentary behaviour and physical activity are associated with biomarkers of endothelial dysfunction and low-grade inflammation-relevance for (pre)diabetes: the Maastricht study. *Diabetologia* **65** (5), 777–789 (2022). 35119485 10.1007/s00125-022-05651-3 PMC8960649

[21] Islam, M. M., Satici, M. O. & Eroglu, S. E. Unraveling the clinical significance and prognostic value of the neutrophil-to-lymphocyte ratio, platelet-to-lymphocyte ratio, systemic immune-inflammation index, systemic inflammation response index, and delta neutrophil index: an extensive literature review. *Turk. J. Emerg. Med.* **24** (1), 8–19 (2024). 38343523 10.4103/tjem.tjem_198_23 PMC10852137

[22] Samad, F. & Ruf, W. Inflammation, obesity, and thrombosis. *Blood* **122** (20), 3415–3422 (2013). 24092932 10.1182/blood-2013-05-427708 PMC3829115

[23] Uribe-Querol, E. & Rosales, C. Neutrophils actively contribute to obesity-associated inflammation and pathological complications. *Cells* ;**11**(12). (2022). 10.3390/cells11121883 PMC9221045 35741012

[24] Santilli, F., Vazzana, N., Liani, R., Guagnano, M. T. & Davì, G. Platelet activation in obesity and metabolic syndrome. *Obes. Rev.* **13** (1), 27–42 (2012). 21917110 10.1111/j.1467-789X.2011.00930.x

[25] Wang, P. et al. Monocyte-to-high-density lipoprotein ratio and systemic inflammation response index are associated with the risk of metabolic disorders and cardiovascular diseases in general rural population. *Front. Endocrinol. (Lausanne)*. **13**, 944991 (2022). 36157453 10.3389/fendo.2022.944991 PMC9500229

[26] Liu, Z. & Zheng, L. Associations between SII, SIRI, and cardiovascular disease in obese individuals: a nationwide cross-sectional analysis. *Front. Cardiovasc. Med.* **11**, 1361088 (2024). 39238504 10.3389/fcvm.2024.1361088 PMC11374596

[27] Frasca, D., Diaz, A., Romero, M., Vazquez, T. & Blomberg, B. B. Obesity induces pro-inflammatory B cells and impairs B cell function in old mice. *Mech. Ageing Dev.* **162**, 91–99 (2017). 28111127 10.1016/j.mad.2017.01.004 PMC5560850

[28] Gregor, M. F. & Hotamisligil, G. S. Inflammatory mechanisms in obesity. *Annu. Rev. Immunol.* **29**, 415–445 (2011). 21219177 10.1146/annurev-immunol-031210-101322

[29] Karelis, A. D. et al. The metabolically healthy but obese individual presents a favorable inflammation profile. *J. Clin. Endocrinol. Metab.* **90** (7), 4145–4150 (2005). 15855252 10.1210/jc.2005-0482

[30] Yang, Y. L. et al. Systemic immune-inflammation index (SII) predicted clinical outcome in patients with coronary artery disease. *Eur. J. Clin. Invest.* **50** (5), e13230 (2020). 32291748 10.1111/eci.13230

[31] Dziedzic, E. A. et al. Investigation of the associations of novel inflammatory biomarkers-systemic inflammatory index (SII) and systemic inflammatory response index (SIRI)-With the severity of coronary artery disease and acute coronary syndrome occurrence. *Int. J. Mol. Sci.* **23**(17). (2022). 10.3390/ijms23179553 PMC9455822 36076952

[32] Chen, Y., Xie, K., Han, Y., Xu, Q. & Zhao, X. An Easy-to-Use nomogram based on SII and SIRI to predict in-Hospital mortality risk in elderly patients with acute myocardial infarction. *J. Inflamm. Res.* **16**, 4061–4071 (2023). 37724318 10.2147/JIR.S427149 PMC10505402

[33] Xia, Y. et al. Systemic immune inflammation index (SII), system inflammation response index (SIRI) and risk of All-Cause mortality and cardiovascular mortality: A 20-Year Follow-Up cohort study of 42,875 US adults. *J. Clin. Med.* ;**12**(3). (2023). 10.3390/jcm12031128 PMC9918056 36769776

[34] Hamilton, M. T., Hamilton, D. G. & Zderic, T. W. Sedentary behavior as a mediator of type 2 diabetes. *Med. Sport Sci.* **60**, 11–26 (2014). 25226797 10.1159/000357332 PMC4364419

[35] Lumeng, C. N., Bodzin, J. L. & Saltiel, A. R. Obesity induces a phenotypic switch in adipose tissue macrophage polarization. *J. Clin. Invest.* **117** (1), 175–184 (2007). 17200717 10.1172/JCI29881 PMC1716210

[36] Wunderlich, F. T. et al. Interleukin-6 signaling in liver-parenchymal cells suppresses hepatic inflammation and improves systemic insulin action. *Cell. Metab.* **12** (3), 237–249 (2010). 20816090 10.1016/j.cmet.2010.06.011

[37] Konstantinides, S., Schäfer, K., Koschnick, S. & Loskutoff, D. J. Leptin-dependent platelet aggregation and arterial thrombosis suggests a mechanism for atherothrombotic disease in obesity. *J. Clin. Invest.* **108** (10), 1533–1540 (2001). 11714745 10.1172/JCI13143 PMC209418

[38] Ohashi, K. et al. Adiponectin promotes macrophage polarization toward an anti-inflammatory phenotype. *J. Biol. Chem.* **285** (9), 6153–6160 (2010). 20028977 10.1074/jbc.M109.088708 PMC2825410

[39] Ouchi, N., Parker, J. L., Lugus, J. J. & Walsh, K. Adipokines in inflammation and metabolic disease. *Nat. Rev. Immunol.* **11** (2), 85–97 (2011). 21252989 10.1038/nri2921 PMC3518031

[40] Wellen, K. E. & Hotamisligil, G. S. Obesity-induced inflammatory changes in adipose tissue. *J. Clin. Invest.* **112** (12), 1785–1788 (2003). 14679172 10.1172/JCI20514 PMC297006

[41] Li, S. et al. The association of sedentary time with sleep disturbances among the US population, 2005 to 2014. *BMC Public. Health*. **24** (1), 2565 (2024). 39300368 10.1186/s12889-024-20114-7 PMC11414297

[42] Ryu, S. et al. Relationship of sitting time and physical activity with non-alcoholic fatty liver disease. *J. Hepatol.* **63** (5), 1229–1237 (2015). 26385766 10.1016/j.jhep.2015.07.010

