# Supplementary material for: Association between novel inflammatory biomarkers SII, SIRI, and obesity in sedentary adults: NHANES 2007–2020
Source: Sci Rep. 2025 Jul 1;15:22300. doi: 10.1038/s41598-025-08121-z (PMC12215839; doi:10.1038/s41598-025-08121-z)
Supplement: Supplementary file 1 — Supplementary Material 1 [file 41598_2025_8121_MOESM1_ESM.docx]

Supplement Table S1: The mean, median, and standard deviation of all numerical variables.

| **Variable** | **ST<5h (n=5426)** | | |  | **ST>=5h** (n=7283) | | |
| --- | --- | --- | --- | --- | --- | --- | --- |
|  | **Mean** | **Median** | **SD** |  | **Mean** | **Median** | **SD** |
| **Age** (years) | 49.59 | 50.00 | 17.16 |  | 50.65 | 51.00 | 17.99 |
| **SII** (1000 cells/μl) | 509.96 | 437.82 | 484.41 |  | 533.62 | 453.14 | 344.12 |
| **l**nSII (1000 cells/μl) | 6.08 | 6.08 | 0.54 |  | 6.12 | 6.12 | 0.56 |
| **SIRI** (1000 cells/μl) | 1.11 | 0.91 | 0.84 |  | 1.23 | 0.99 | 0.94 |
| **TC**  (mmol/L) | 4.96 | 4.89 | 1.05 |  | 4.87 | 4.78 | 1.05 |
| **HDL**-C (mmol/L) | 1.41 | 1.34 | 0.41 |  | 1.38 | 1.32 | 0.41 |
| **TG (**mmol/L) | 1.30 | 1.13 | 0.72 |  | 1.32 | 1.13 | 0.75 |
| **LD**L-C (mmol/L) | 2.96 | 2.90 | 0.92 |  | 2.88 | 2.82 | 0.92 |

SII: Systemic Immune-Inflammation Index

SIRI: Systemic Inflammation Response Index

lnSII: The natural logarithm of the systemic immune inflammatory index.

TC: total cholesterol; HDL-C: high-density lipoprotein; TG: triglyceride; LDL-C: Low density lipoprotein-cholesterol.
